# Feelings toward lying flatism and attitudes toward singlehood: the mediating role of happiness belief

**DOI:** 10.1186/s40359-023-01187-2

**Published:** 2023-05-12

**Authors:** Zeng-Qing Heng, Joey Chin, Chee-Seng Tan

**Affiliations:** Department of Psychology and Counselling, Faculty of Arts and Social Science, Universiti Tunku Abdul Rahman (UTAR), Jalan Universiti, Bandar Barat, Kampar, 31900 Perak D.R. Malaysia

**Keywords:** Lying flatism, Happiness belief, Attitudes toward singlehood, Singlism, Fear of being single, Malaysia, Experimental design

## Abstract

**Background:**

Lying flatism, a new emerging lifestyle by refusing to participate in consumerist lifestyles, is anticipated to be related to singlehood. Based on the Theory of Reasoned Action, we proposed a mediation model to examine the indirect relationship between feelings towards lying flatism and attitudes toward singlehood via individuals’ belief in that happiness can be achieved without romantic relationships (happiness belief).

**Methods:**

Using purposive and snowball sampling methods, 232 single Malaysian young adults participated in an online experiment consisting of a writing task (to manipulate feelings toward lying flatism), single-item measures of manipulation checking and happiness belief, Attitudes toward Singlehood Scale, Negative Stereotyping of Single Persons Scale (a measure of singlism) and Fear of Being Single Scale.

**Results:**

T-Test results support the writing task that successfully induced positive feelings toward lying flatism. Mediation analysis showed that the feelings toward lying flatism measured before the writing task, but not the manipulation of lying flatism, has an indirect relationship with attitudes toward singlehood via happiness belief, after controlling for gender, singlism, and fear of being single.

**Conclusions:**

The findings offer preliminary support to the hypothetical relationships among feelings towards lying flatism, happiness belief, and attitudes toward singlehood. Implications of the findings are discussed.

Marriage is no longer perceived as a universal life goal [1–3] since singlehood has become a favorable lifestyle worldwide. A stark singlehood is seen among young adults, especially in the United States [4], Singapore [5], South Korea [6], and Japan [7]. Asia Pacific recorded the highest number of single citizens [8]. One possible reason for such high singlehood population is that young people have started to embrace different lifestyles and perspectives such as low desirability in Japan; the Sippo generation in Korea; Not in Education, Employment, or Training (NEET) in the United Kingdom; and the Boomerang Generation in the United States in response to the tough competition of surviving in society [9].

A similar trend has also been documented in China. Young advocators of lying flatism (躺平主义, pinyin: tǎng píng zhǔ yì) portray a blameless and silent protest against the unfairness of society whereby personal efforts can no longer be effective to alter the industrial and structural factors [9]. These youngsters refuse to participate in consumerist lifestyles such as going to work, purchasing for their material desire, involving in marriage or having children [10]. Hence, it is reasonable to assume that individuals who have positive feelings toward lying flatism are likely to have a positive attitudes toward singlehood. This assumption, however, has not been empirically tested in any context. Furthermore, how lying flatism is related to attitudes toward singlehood also remains unknown. As lying flatists tend to live without marriage or romantic relationships [11] to avoid the pressure of modern urban life [10], they believe that happiness could be achieved without a romantic relationship (hereinafter referred to as happiness belief). Therefore, it is believed that happiness belief plays a mediator role in the relationship between feelings toward lying flatism and attitudes toward singlehood.

The hypothetical mediation model is congruent with the Theory of Reasoned Action (TRA) [12, 13]. According to the TRA, behavior is primarily predicted by an individual’s intention to carry out that behavior. This intention is influenced by two components: attitudes and subjective norms, which operate through separate routes. Attitudes toward behavior are shaped by an individual’s beliefs about the consequences of the behavior (i.e., behavioral beliefs), while subjective norms are influenced by an individual’s perception of the expectations of significant others (i.e., normative beliefs). Additionally, background factors such as emotions and education play a role as antecedent factors in shaping behavioral and normative beliefs [14]. In this study, we focused on the pathway from background factors to behavioral beliefs to attitudes towards behavior. Specifically, we used feelings towards lying flatism, happiness beliefs, and attitudes toward singlehood as proxies for the background factor, behavioral beliefs, and attitudes toward behavior, respectively, to construct the hypothetical mediation model.

In the remaining sections, we first reviewed the concept of lying flatism, attitudes toward singlehood, and their relationship. Then, we reviewed happiness belief and its relationship with feelings toward lying flatism and attitudes toward singlehood respectively and provided an overview of the present study. Finally, we presented the methodology of the study and analysis results and discussed the findings and their implications.

## Lying flatism

Lying flatism is a trending philosophy introduced in an April 2022 online post named “Lying down flat signifies justice (躺平即是正义)” on Baidu Tieba, a mainland China forum [15, 16]. Resulted from an anti-consumerism movement [16], lying flatism refers to a state of living in which individuals are not having desires in their life, not focusing on anything, and are not willing to move forward [17]. People who practice lying flatism (or lying flatists) reduce their materialistic desires, refuse job promotions, disengage in romantic relationships, deny house or car purchases, and forgo marriage and children [18, 19]. Instead of working hard in fighting for better lives, lying flatists only wish to maintain the lowest possible standards of living, focus on their feelings, and live in their current moments [16, 20, 21].

The emergence of the lying flatism can attribute to two reasons. First, lying flatism is attributed to the severe working overtime culture in the China Industry [22]. For instance, China’s technology workers have a well-known extreme overtime system named 996: working for 12 h per day (from 9 am to 9 pm) and 6 days per week [23]. This hustle culture imposes intense pressure on the employees. The workers relentlessly feel exhausted and frustrated with the working system, and hence start practicing lying flatism to escape from the stressful circumstances and to take rests. Second, individuals adopt lying flatism to protest silently against the feeling of unfairness [9]. Lying flatism is recognized as a non-violent way of resisting the capital system and exploitation by capitalists [17]. It is essential to note that lying flatism is conceptually different from minimalist lifestyle, which revolves around utilizing only items that have a practical purpose, adopting a simple way of life, and possessing only what is essential for one’s daily routines. On the contrary, individuals lie flat to have control of their destiny and not be a money-making machine for capitalists [9].

## Singlehood

Singlehood refers to a state of being unmarried or not involved in a romantic relationship [24, 25] and has been assumed to be an attitude and expression of choice to stay single [1]. Apostolou [26] has discussed individuals’ singlehood decisions using the fitness-increasing strategy. Individuals are willing to stay single and invest in themselves for their improvement, thus becoming more attractive and having better qualities in attaining better mates in the future mating market. Apostolou and colleagues [27] have also concluded several reasons for being single. The most common factors include poor flirting skills, retaining one’s freedom, fear of being hurt, owning different priorities, and being too picky. Moreover, men are more likely to stay single as they desire the freedom to flirt, while women stay single to avoid getting hurt besides perceiving themselves as undesirable mate. Similar findings are also documented in bachelorhood research. For example, both male and female bachelors associated bachelorhood with freedom, independence, and psychological stability [28], while single men who gave up involvement in a romantic relationship in China because of their physical appearance and income [29].

### Attitudes toward singlehood (AtS)

A romantic relationship is the most crucial relationship among most adults [30]. Individuals engaging in romantic relationships or marriage have higher well-being (e.g., happiness, life satisfaction, positive affect) and lower distress compared to singles [3, 31–35]. On the contrary, the absence of companionship is a common cause of individuals’ fear of being single [36], which is negatively correlated with one’s well-being [1].

However, it has been found that staying single can be beneficial to individuals’ well-being [3, 37]. Individuals who are always single can be as happy as individuals who are currently married [38]. Indeed, single individuals can cultivate higher life satisfaction [39] and healthier mental conditions [40] by maintaining a high quality of social connection [25]. Single individuals have better control over their time as they can focus on their schedules without worrying about their partners’ [41, 42]. Moreover, single individuals are free from maintaining their partners’ expectations to keep attracting their romantic partners [43]. They can also save their money and enjoy the freedom to control their finance [44, 45].

Meanwhile, the inconsistent findings of the relationship between singlehood and well-being could be due to people’s choice to stay single (i.e., involuntary vs. voluntary singlehood). Involuntary singlehood refers to people experiencing difficulties in attracting a partner due to poor qualities [46], while voluntary singlehood refers to people being single of their own will [1]. To better understand people’s view of singlehood, Tan and colleagues [37] developed the Attitudes toward Singlehood Scale (AtSS) to examine people’s attitudes toward singlehood from the affective, behavioral, and cognitive aspects. Tan and colleagues found that individuals who have positive attitudes toward singlehood tend to have a higher level of life satisfaction and well-being.

## The relationship between lying flatism and attitudes toward singlehood

It is assumed that lying flatism has a positive relationship with attitudes toward singlehood. According to News qq [47], more and more young adults are choosing to stay single and insisting on the “lying-flat view of romantic relationships (躺平式恋爱观)”. They are satisfied with their singlehood status and prefer to maintain calm and plain lifestyles to avoid fearfulness when people approach them and interrupt their lives [16, 47], or it could result from anxiety brought by past unsuccessful relationships [48]. A similar pattern has also been witnessed in Japan. Japanese with the mindset of lying flatism namely the Satori generation (さとり世代, Satori Sedai) do not strive to earn more money, but instead choose to live modestly (e.g., valuing simple pleasures), distance themselves from mainstream values of consumerism, prioritize a pursuit of happiness rooted in simplicity, and are not interested in romantic relationships [49, 50]. It is claimed that the latter could promote the development of positive attitudes toward singlehood [41].

## The mediating role of happiness belief

As reviewed, lying flatism is assumed to have a positive relationship with attitudes toward singlehood. However, it is believed that there is an indirect relationship between the two constructs. In this section, we reviewed the theoretical and empirical rationales for the hypothetical mediating role of happiness belief in the association between feelings toward lying flatism and attitudes toward singlehood.

### The relationship between lying flatism and happiness belief

There are three reasons to assume a positive relationship between lying flatism and the belief that happiness can be achieved without a romantic relationship. First, individuals who follow the lying flatism perspective prefer lives with minimum spending [11]. To achieve that goal, they avoid having romantic relationships, marriage, and children [11, 21] which will cost them some resources like time and money [26]. In other words, staying single helps lying flatists to control their finance [43] by reducing expenses (i.e., retaining more resources for themselves) and living a minimum-spending lifestyle. Consequently, they can enjoy their lives [20], feel blessed by their current condition [51], and experience happiness in a different way [16].

Second, staying single leads lying flatists to experience happiness by having a sense of control. In certain societies such as China and Malaysia, success in life is defined as getting married and having children [20, 52], while staying single represents a social failure [53]. Attaining romantic relationships, marriage, and children are always demanded not only by the parents but also by members of society [41]. However, lying flatists are more settled with living lives in their ways instead of fulfilling the social demand [15, 17, 54]. When lying flatists can control their lives by defying social demands (i.e., engaging in a romantic relationship), they are living joyously because individuals who possess a higher ability to control life events are associated with superior well-being [55]. Finally, engaging or retaining a romantic relationship can be stressful [56] and detrimental to one’s happiness [57, 58]. By avoiding overwhelming stress [59], they could feel relieved without worrying about the end of a romantic relationship in them [54].

Taken together, individuals who have positive feelings toward lying flatism are likely to believe that their happiness can be achieved without engaging in a romantic relationship as they can reserve resources, have higher control, and reduce stress when staying single.

### The relationship between happiness belief and attitudes toward singlehood

Literature (e.g., [38, 60]) has shown that single individuals can be as happy and healthy as their married counterparts. The belief in happiness without engaging in romantic relationships can be positively associated with positive attitudes toward singlehood among single people contented with their current lifestyles. The association could result from two reasons. First, single people can obtain a sense of belonging from supportive social networks (e.g., friends, and family) other than romantic relationships. The intimate connections with friends and family not only promote single individuals’ well-being [39, 40] but also reduce their loneliness in the absence of romantic relationships [25]. Second, as reviewed above, staying single allows people to enjoy benefits such as having more freedom and a less financial burden [43]. Some young adults have different life priorities [26]; they are not interested in getting romantic partners because they can accomplish well-being (e.g., a sense of achievement) by focusing on their career paths [41].

Taken together, people who have supportive social networks and enjoy the benefits of singlehood are likely to believe that well-being can be achieved without romantic relationships and hence, they tend to have positive attitudes toward singlehood [41].

## The present study

Both lying flatism and voluntary singlehood are new and rising trends in the young generation. The present study aimed to expand our understanding of these individual choices by examining whether and how feelings toward lying flatism are related to attitudes toward singlehood. To our best knowledge, measurement of the state-like lying flatism is not available. Hence, we manipulated people’s feelings toward lying flatism using a writing task to understand if having a positive (vs. neutral) view of lying flatism influences one’s happiness belief and attitudes toward singlehood. In addition, we focused on young adults in Malaysia because the proportion of singles has been increasing gradually in Malaysia over the years [53].

The present study adopted a part of the TRA to explain the relationships among feelings toward lying flatism, happiness belief, and attitudes toward singlehood. Specifically, it is hypothesized that (1) feelings towards lying flatism have a positive relationship with happiness belief, (2) feelings toward lying flatism have a positive relationship with attitudes toward singlehood, (3) happiness belief is positively associated with attitudes toward singlehood, (4) happiness belief mediates the relationship between feelings toward lying flatism and attitudes toward singlehood. In addition, we statistically controlled for three variables (i.e., singlism, fear of being single, and gender) to get rid of their confounding effects on the relationships among the three target variables.

The present study is expected to shed light on the inquiry of whether and how lying flatism plays a role in shaping people’s positive attitudes toward singlehood. The findings will not only broaden our understanding of lying flatism but may also offer insights into the negative impact of lying flatism on the birth rate.

## Method

### Participants and research design

The present study adopts a quantitative cross-sectional research design. According to Xin [9], lying flatism is embraced by generation Z who were born between 1997 and 2012 (i.e., aged 9 to 24 in 2021). Hence, individuals aged 18 to 24 served as the target population of the present study as lying flatists are mostly composed of individuals within this age range [61]. Purposive and snowball sampling methods were used to recruit participants who fulfilled the following selection criteria: (a) aged between 18 and 24 years old, (b) single (i.e., currently not in a romantic relationship), and (c) Malaysian who is currently residing in Malaysia.

According to the Monte Carlo Power Analysis for Indirect Effects [62], a minimum of 225 participants are needed to achieve a power of 0.95. Although a total of 522 responses were recorded, 290 responses were omitted from the data analysis due to failure in the attention checking item (90 cases), violation of the inclusion criteria (68 cases), not answering the survey except for the demographic Sect. (62 cases), violation of essay instruction (40 cases), not giving consent to participate (25 cases), not answering one of the measurements (4 cases), and univariate outlier (1 case). The remaining 232 successful responses were retained and submitted for further data analysis.

The sample consisted of 139 females (59.9%) and 93 males (40.1%) with ages ranging from 18 to 24 (*M =* 21.37, *SD* = 1.332). In terms of ethnicity, the majority were Chinese (86.6%), followed by Indians (6.9%), Malays (5.6%), and other ethnic groups (0.9%). Most of the participants never engaged in a romantic relationship (62.5%). All the participants were currently single (not in a romantic relationship) with the highest singlehood duration being less than 3 months (53.4%). In terms of working status, the majority of the participants were currently studying (88.4%), 19 participants (8.2%) were currently employed, three participants (1.3%) were undergoing internship, working as a freelancer, and waiting for a job offer, four participants were looking for a job (1.7%), and one missing value.

### Instruments

The English version of the following instruments was used in the present study for data collection.

#### Manipulation of the feelings toward lying flatism and checking

Individuals’ feelings toward lying flatism were experimentally manipulated in the present study using a writing task, which could be easily completed and is effective to manipulate self-awareness [63]. Participants were randomly assigned to the experimental group or control group. Then, all participants read a brief introduction about lying flatism and the instruction of writing an essay on the given topic.

To induce positive feelings toward lying flatism, participants in the experimental group were instructed to write an essay to indicate their support of lying flatism. Three sample statements regarding lying flatism: *(1) Practising lying flatism allows individuals to stay away from stressful circumstances; (2) Lying flatism allows us to live in the current movement and focus on our states of feelings; (3) Practising lying flatism can avoid pressure and expectations of family, friends, and society*, were provided to help participants to understand the writing task.

On the other hand, participants in the control group were assigned to write an essay on a neutral topic “weather”. Similarly, they were provided with three questions as references to write about the topic. The questions and statements provided were *(1) How is the weather today? Was it the same as yesterday’s? (2) In which month has the most comfortable weather occurred in your hometown? (3) Share with us any interesting myths about the weather.*

A single-item self-report (“*To what extent are you feeling positive toward lying flatism?*”) was created to examine the effectiveness of the writing task in manipulating an individual’s feelings toward lying flatism. All participants answered twice on the single item using a 5-point Likert point from 1 *(very negative)* to 5 *(very positive)*: before the commencement of the writing task (i.e., pre-manipulation measure) and right after the completion of the writing task (i.e., post-manipulation measure). Individuals who score high tend to have positive feelings toward lying flatism. Moreover, the manipulation is considered effective if the post-manipulation measured score is higher than the pre-manipulation measured score and if the difference is statistically significant.

#### Happiness belief

We adapted and integrated the measures of the belief that happiness can be achieved without marriage for men and women [25] into a single-item measure of happiness belief. Participants rated the item “I believe that I can have full and happy lives without a romantic relationship” on a scale from 1 *(strongly disagree)* to 7 *(strongly agree)*. The higher the score, the stronger the belief that happiness can be achieved without a romantic relationship.

#### Attitudes toward singlehood scale

The Attitudes toward Singlehood Scale (AtSS) was used to measure the attitudes of young adults toward singlehood [37]. The AtSS consists of 9 items with 3 items each for the affective, behavioral, and cognitive subscales. Sample items are *I feel happy when I am single* (affective subscale), *I stay single to have more personal space* (behavioral subscale), and *Engaging in a romantic relationship is not important* (cognitive subscale). Participants used a 7-point Likert point (1: strongly disagree; 7: strongly agree*)* to indicate the extent to which they agreed with the items. A higher (average) score indicates higher participants’ positive attitudes toward singlehood, and vice versa. The AtSS showed excellent reliability in the present study with Cronbach alpha (α) = 0.91, McDonald omega (ω) = 0.91, and good validity for Malaysian young adults [37].

### Control variables

#### Singlism

Singlism refers to the negative stereotype and discrimination toward individuals who are staying single [64]. The 30-item Negative Stereotyping of Single Persons Scale (NSSP) [65] was used in the present study to measure an individual’s anti-singles attitude using a 7-point Likert scale ranging from 1 (*strongly disagree*) to 7 (*strongly agree*). A higher average score represents a greater stereotype. The NSSP demonstrated excellent reliability in the present study (α = 0.91, ω = 0.91).

#### Fear of being single

Fear of being single refers to a state of being anxious or distressed in the absence of a romantic partner or when staying single [36]. People who fear being single demand romantic partners as opposed to lying flatists who prefer single, calm, and plain lifestyles [16]. It is therefore reasonable to assume that fear of being single may confound the relationship between lying flatism and attitudes toward singlehood.

A 6-item Fear of Being Single Scale (FBSS) was employed to measure the extent to which individuals fear being single [36]. Participants indicated their agreement with the items using a 5-Likert point from 1 *(not at all true)* to 5 *(very true)*. Higher (total) scores indicate a higher level of fear. The FBSS showed satisfactory reliability in the present study (α = 0.79, ω = 0.79).

#### Gender

Tan and colleagues [34] found gender differences in attitudes toward singlehood scores. Male participants were found to have a lower score than female participants. Hence, gender was included as a control variable in the present study to exclude the impacts of gender differences.

### Research procedure

The study was conducted online due to the COVID-19 pandemic. An online survey was created using Qualtrics and then distributed through several social media platforms (e.g., Instagram, and Facebook). The data collection was conducted from 20 to 2021 to 11 November 2021.

After giving their consent, participants responded to the measures in the following sequence: demographic, NSSP and FBSS, the pre-manipulation measure of feelings toward lying flatism, essay writing task, the post-manipulation measure of feelings toward lying flatism, Happiness Belief, and AtSS. Participants were debriefed at the end of the study. Participants who completed the survey and passed the attention-checking items received a token of RM 5.00. The procedure was approved by the university’s Scientific and Ethical Review Committee (Ref: U/SERC/196/2021).

### Analytical strategy

IBM SPSS version 21 was used to carry out the statistical analyses. A mixed analysis of variance (ANOVA) with time (pre- vs. post-measured) as the within-subject factor, group condition (experimental vs. control group) as the between-subject factor, and feelings toward lying flatism as the outcome variable was conducted to measure if the written task manipulation is effective in inducing participants’ positive feelings toward lying flatism. The proposed mediation model was tested using Model 6 of the PROCESS SPSS Macro ver. 4.0 [66]. The mediating effect was examined with 10,000 bootstrapping and 95% percentile confidence intervals (CI). The effect is statistically significant if the CI does not include zero.

## Results

### Manipulation checking

The results of mixed ANOVA showed a significant within-subject effect of time and an interaction effect of time and group. The between-subject effect of the group was not significant. Simple effect tests were then conducted to clarify the interaction effect. Specifically, two independent sample t-Tests were first conducted to test the effectiveness of the writing task in manipulating participants’ feelings toward lying flatism. The first t-Test compared the pre-manipulation measured feelings toward lying flatism score between the experimental and control groups. Levene’s test was not statistically significant, *F*(1,230) = 1.16, *p* = .282, indicating the assumption of equal variance was not violated. Results showed no significant difference between the experimental group (*M* = 2.83, *SD* = 0.90) and the control group (*M* = 2.86, *SD* = 0.96), *t*(230) = 0.28, *p* = .778.

The second t-Test compared the feelings toward lying flatism score measured after the writing task. Levene’s test was not significant, *F*(1,230) = 1.00, p = .317. However, there was a significant difference between the two groups, *t*(230) = -2.11, *p* = .036, Cohen’s d = 0.28 95% CI [0.02, 0.54]. The experimental group (*M* = 3.15, *SD* = 0.89) reported a higher score than their counterparts in the control group (*M* = 2.88, *SD* = 0.98).

We also conducted a paired sample t-Test to further examine the effectiveness of the essay writing task by comparing the pre- and post-measured feelings toward lying flatism of the experimental group. Results showed a significant difference: *t*(115) = 4.79, *p* < .001, *d* = 0.45 95% CI [0.25, 0.63]. Specifically, after completing the essay writing task, participants reported a higher score (*M* = 3.15, *SD* = 0.89) than their baseline score (*M* = 2.83, *SD* = 0.90). The consistent results indicate that the manipulation is effective.

### Mediation analysis

The writing task successfully manipulated participants’ feelings toward lying flatism. Drawing from the Theory of Reasoned Action, the induced feeling is hypothesized to have an indirect impact on attitudes toward singlehood via happiness belief. Before testing the hypothetical mediation model, we examined the descriptive statistics and correlations for the target variables (see Table 1).

**Table 1:** Descriptive Statistics and Intercorrelation for Target Variables

	1	2	3	4	5	6
1. Gender	1					
2. Singlism	− 0.30***	1				
3. Fear of being single	− 0.13*	0.55***	1			
4. Manipulation	0.06	0.02	0.06	1		
5. Happiness Belief	0.24***	− 0.42***	− 0.43***	0.01	1	
6. Attitudes_Singlehood	0.30***	− 0.46***	− 0.43***	− 0.01	0.70***	1
Minimum	0	1.10	6	0	1	2.11
Maximum	1	4.477	28	1	7	7
Mean	N/A	2.98	14.47	N/A	4.94	5.07
SD	N/A	0.77	5.31	N/A	1.46	1.00
Skewness	N/A	-0.06	0.30	N/A	-0.38	-0.17
Kurtosis	N/A	-0.67	-0.56	N/A	-0.57	-0.38

*Note*. *N* = 232.

Gender: 0 = male, 1 = female; Manipulation: 0 = control group, 1 = experimental group; lying flatism; Attitudes_Singlehood = Attitudes toward singlehood; N/A : Not applicable.

Standard Error of skewness = 0.16; Standard Error of kurtosis = 0.32.

* *p* < .05; *** *p* < .001.

Pearson’s correlation analysis showed that the manipulation of feelings toward lying flatism (dummy coded: 0 = control group, 1 = experimental group) did not correlate with any of the variables. Happiness belief, on the other hand, had a positive relationship with gender (dummy coded: 0 = male, 1 = female) and attitudes toward singlehood besides a negative relationship with singlism and fear of being single. Finally, attitudes toward singlehood were positively associated with gender and negatively associated with singlism and fear of being single.

Next, we examined the proposed mediation model with the group condition (0: control group; 1: experimental group) as the predictor variable, happiness belief as the mediator, and attitudes toward singlehood as the outcome variable. We included gender, singlism, and fear of being single as covariate variables because the three variables were correlated with happiness belief and attitudes toward singlehood respectively.

Table 2 shows the summary of the mediation analysis (unstandardized) results. The effect of group conditions on happiness belief was not significant. Similarly, group conditions did not influence attitudes toward singlehood in the total effect model and direct effect model. Happiness belief (*B* = 0.39, *p* < .001), on the other hand, was positively correlated with attitudes toward singlehood. Finally, the mediating effect of happiness belief was not significant.

**Table 2:** Summary of Mediation Analysis Results with Group Condition as the Predictor

	*B*	*SE*	*t*	*p*	95% CI
*Outcome: Happiness belief*					
Group	0.06	0.17	0.37	0.715	-0.27, 0.39
Gender	0.39	0.18	2.17	0.031	0.04, 0.75
Singlism	-0.43	0.14	-3.14	0.002	-0.69, -0.16
Fear of being single	-0.08	0.02	-4.11	< 0.001	-0.12, -0.04
*Outcome: Attitudes toward singlehood (Direct effect model)*					
Group	-0.03	0.09	-0.29	0.773	-0.21, 0.15
Happiness Belief	0.39	0.04	10.94	< 0.001	0.32, 0.46
Gender	0.23	0.10	2.29	0.023	0.03, 0.42
Singlism	-0.18	0.07	-2.35	0.020	-0.32, -0.03
Fear of being single	-0.02	0.01	-1.66	0.099	-0.04, 0.003
*Outcome: Attitudes toward singlehood (Total effect model)*					
Group	-0.002	0.11	-0.02	0.985	-0.22, 0.22
Gender	0.38	0.12	3.16	0.002	0.14, 0.61
Singlism	-0.34	0.09	-3.79	< 0.001	-0.52, -0.16
Fear of being single	-0.05	0.01	-3.81	< 0.001	-0.07, -0.02
*Indirect effect*	0.02	0.07	-	-	-0.11, 0.15

*Note*. *B* = Unstandardized regression coefficient; *SE* = Standard error; *CI* = Confidence interval; Group: 0 = control group, 1 = experimental group; Gender: 0 = Male, 1 = Female; *Indirect effect* = Manipulation→Happiness Belief→Attitudes toward singlehood.

### Exploratory analysis

The mediation analysis showed that happiness belief does not mediate the effect of lying flatism manipulation on attitudes toward singlehood. It was also found that the manipulation of feelings toward lying flatism did not influence happiness belief and attitudes toward singlehood respectively. One possibility is that the effect of the manipulation task is too small, and our sample size is insufficient to detect its impact. To clarify the relationships between the three variables, we conducted another mediation analysis with the feelings toward lying flatism measured before the writing manipulation task, which represents the participants’ initial feelings toward lying flatism, as the predictor. Before that, we conducted a Pearson correlation analysis to understand if the pre-measured feelings toward lying flatism are associated with the target and controlled variables. Results showed that the pre-measured feelings toward lying flatism had a positive relationship with happiness belief (*r* = .281, *p* < .001) and attitudes toward singlehood (*r* = .329, *p* < .001), but did not have a relationship with singlism and fear of being single, respectively.

Table 3 summarizes the unstandardized results of the mediation model. The pre-measured feelings toward lying flatism were found to have a positive relationship with happiness belief (*B* = 0.36, *p* < .001) and attitudes toward singlehood in the total effect model (*B* = 0.30, *p* < .001) and direct effect model (*B* = 0.17, *p* < .001). Likewise, there was a positive relationship between happiness belief and attitudes toward singlehood (*B* = 0.36, *p* < .001). Finally, the mediating effect of happiness belief was found significant: B = 0.13, SE = 0.04, 95% CI [0.06, 0.20].

**Table 3:** Summary of Mediation Analysis Results with Pre-Measured Lying Flatism as the Predictor

	*B*	*SE*	*t*	*p*	95% CI
*Outcome: Happiness belief*					
Pre-Lying Flatism	0.36	0.09	4.10	< 0.001	0.19, 0.54
Gender	0.41	0.17	2.38	0.018	0.07, 0.75
Singlism	-0.40	0.13	-3.02	0.003	-0.66, -0.14
Fear of being single	-0.07	0.02	-3.97	< 0.001	-0.11, -0.04
*Outcome: Attitudes toward singlehood (Direct effect model)*					
Pre-Lying Flatism	0.17	0.05	3.38	< 0.001	0.07, 0.27
Happiness Belief	0.36	0.04	9.92	< 0.001	0.29, 0.43
Gender	0.24	0.10	2.55	0.012	0.06, 0.43
Singlism	-0.18	0.07	-2.42	0.016	-0.32, -0.03
Fear of being single	-0.02	0.01	-1.75	0.082	-0.04, 0.002
*Outcome: Attitudes toward singlehood (Total effect model)*					
Pre-Lying Flatism	0.30	0.06	5.19	< 0.001	0.19, 0.41
Gender	0.39	0.11	3.47	< 0.001	0.17, 0.61
Singlism	-0.32	0.09	-3.73	< 0.001	-0.49, -0.15
Fear of being single	-0.04	0.01	-3.70	< 0.001	-0.07, -0.02
*Indirect effect*	0.13	0.04	-	-	0.06, 0.20

*Note*. *B* = Unstandardized regression coefficient; *SE* = Standard error; *CI* = Confidence interval; Pre-Lying Flatism: Pre-measured feelings toward lying flatism; Gender: 0 = Male, 1 = Female; *Indirect effect* = Pre-measured feelings toward lying flatism→Happiness Belief→Attitudes toward singlehood.

## Discussion

Singlehood is a favorable lifestyle in both Western and Asia regions [8, 50]. The present study proposed and examined a mediation model to illuminate the relationships among the feelings toward lying flatism, happiness belief, and attitudes toward singlehood of young adults in Malaysia. Although the hypothetical mediating role of happiness belief is not supported, several interesting findings are revealed.

In contrast to our hypothesis, happiness belief did not mediate the impact of feelings toward lying flatism on attitudes toward singlehood. Inspection of the results found that the manipulation of feelings toward lying flatism did not have any impacts on happiness belief and attitudes toward singlehood respectively. As mentioned above, the results could be attributed to three reasons: (a) the manipulation task is not effective, (b) feelings toward lying flatism do not have a relationship with the two constructs, and (c) the induced effect is too small, and the sample size of the current study is insufficient to detect the effect. Among them, the third reason seems more plausible. This is because our manipulation checking indicates that the participants in the experimental group reported a higher score in feelings toward lying flatism than their counterparts in the control group. Furthermore, the exploratory mediation analysis showed that the pre-measured feelings toward lying flatism are positively associated with both happiness belief and attitudes toward singlehood. Taken together, the results imply that the writing task is effective to manipulate an individual’s feelings toward lying flatism but the induced effect is insufficient to influence happiness belief and attitudes toward singlehood. Future studies, therefore, are suggested to replicate the present study with a larger sample size or another manipulation task.

The (main) mediation analysis also showed a positive relationship between happiness belief and attitudes toward singlehood. It indicates that individuals who believe that romantic relationships are not the only way to achieve happiness are more likely to perceive staying single as advantageous. The finding is in line with the findings that single people can still achieve happiness by developing their sense of belonging and maintaining low levels of loneliness. As a result, they are likely to perceive singlehood positively [2, 25]. Likewise, literature shows that satisfactions from other fulfilling relationships (e.g., supportive family and friends) and important areas of life (e.g., work and leisure) are significant factors of happiness [67]. Individuals who enjoy their lives with supportive social ties other than romantic relationships are satisfied with singlehood [40].

It is intriguing to note that the exploratory analysis (with the feelings toward lying flatism measured before the manipulation task as the predictor) supports the hypothesized mediation model. The findings offer additional insights though they shall be interpreted with caution. First, the positive relationship between the initial feelings toward lying flatism and attitudes toward singlehood indicates that individuals who have positive feelings toward lying flatism are more likely to perceive singlehood as desirable. The result is line with the lying-flat view of a romantic relationship (躺平式恋爱观) whereby individuals who have positive feelings toward lying flatism do not believe in romantic relationships as staying single can be less troublesome (e.g., having more freedom and control over life) than engaging in romantic relationships or marriage [47]. Similarly, feelings toward lying flatism were positively associated with happiness belief, suggesting that individuals who have positive feelings toward lying flatism are more likely to believe that happiness could be achieved without romantic relationships. This is in line with past findings (e.g., [43]) that single individuals can focus more on themselves instead of their romantic partners and that the independence and freedom on allocating their resources could result in true happiness [68]. Indeed, lying flatists have higher control over their life events [20] and this sense of control over self is related to happiness [1]. Finally, consistent with the Theory of Reasoned Action, our results support feelings toward lying flatism (i.e., emotion) having an indirect relationship with one’s attitudes toward singlehood (i.e., attitudes toward the behavior) via happiness belief (i.e., behavioral beliefs). Put differently, individuals who find lying flatism attractive tend to believe that happiness can be achieved from sources other than romantic relationships. Subsequently, people with high levels of happiness belief are likely to hold a positive manner towards singlehood.

### Implications

The present study offers suggestions for the theoretical development of singlehood. First, we demonstrate that the initially measured, but not the manipulated, feelings toward lying flatism, are positively associated with attitudes toward singlehood and happiness belief plays a mediating role in this relationship. Although these exploratory results shall be interpreted with care, the findings reveal a possible direction for future researchers to unveil the underlying process of lying flatism and attitudes toward singlehood. Moreover, the present study lends further support to the Theory of Reasoned Action and extends it to the context of singlehood.

The present study also has some remarkable practical implications. First, the present study successfully manipulated participants’ feelings toward lying flatism using a writing task. The finding not only highlights the effectiveness of writing tasks in influencing people’s feelings but also offers a new methodological direction for future studies on lying flatism. Furthermore, to our best knowledge, our study is the first to examine the roles of feelings toward lying flatism and happiness belief in one’s attitudes toward singlehood. The findings not only reveal new antecedent factors of attitudes toward singlehood on top of the literature (e.g., [37]), but also highlight a potential direction to change one’s attitudes toward singlehood by modifying feelings toward lying flatism and the belief that happiness can be achieved without romantic relationships.

Our findings also lend support to the Theory of Reasoned Action (TRA) by confirming the role of background factors and behavioral beliefs in shaping one’s attitudes toward a behavior. Furthermore, we elucidate the applicability of the theory in the context of singlehood, indicating its potential for understanding the behavior of individuals who choose to remain single.

### Limitations and suggestions

Several limitations of the present study deserve our attention. First, as participants provided personal information (e.g., email address) for verification purposes, the present study is no longer anonymous. Participants might provide answers that seem desirable to avoid judgment due to the social desirability effect [69]. Second, causal relationships among the variables remain open. Although the writing task is effective to manipulate participants’ feelings toward lying flatism, the task does not have any impact on happiness beliefs and attitudes toward singlehood. Likewise, the happiness belief was measured but not experimentally manipulated, it is inappropriate to interpret the findings with a causality perspective.

Apart from that, the present study only focused on single individuals who were currently not in a romantic relationship regardless of their voluntary or involuntary singlehood status. The findings, therefore, are unable to be generalized to people in another relationship status (e.g., married, in a romantic relationship, or cohabitating). With this, we are also unable to examine if there is a difference in attitudes toward singlehood when individuals from the other relationship status group are having positive feelings toward lying flatism, and if the mediating role of happiness belief still holds among people other than single individuals. Finally, feelings toward lying flatism and happiness belief were measured by a single item, which may be seen as questionable or unreliable [70].

To address the above-mentioned limitations, future studies are recommended to use longitudinal design and procedures that can ensure anonymity. The former can furnish a clear picture of the causal relationship between the variables, while the latter can offer a sense of safety for participants to reveal their truthful responses. Moreover, future researchers are also recommended to expand the inclusion criteria to incorporate individuals other than those who are currently single and to replicate the study in other cultural contexts for examining the effectiveness of the writing task in inducing feelings towards lying flatism, as well as the mediating effect of happiness belief in the relationship between feelings toward lying flatism and attitudes toward singlehood. In terms of measurement, future researchers are urged to either develop and employ multi-item measurements or use different approaches (e.g., qualitative method) to capture participants’ feelings toward lying flatism and happiness belief levels in verifying the findings of the present study. In particular, a more comprehensive measurement of happiness belief is urgently needed to address this issue and to clarify the conceptual differences between happiness belief and attitudes toward singlehood. Finally, researchers are also recommended to use double randomization designs [71] (see [72] for example) or half-longitudinal mediation model [73] (see [74] for example) to shed light on the causal relationship between the three target variables.

## Conclusion

Lying flatism is a new lifestyle embraced by young people to cope with the over-competition of limited resources in society [17]. The present study tested a mediation model to clarify the relationship between feelings toward lying flatism, happiness belief, and attitudes toward singlehood among young adults in Malaysia. Our results demonstrated that young adults who find lying flatism favorable tend to believe that happiness can be attained from multiple channels other than romantic relationships. The happiness belief, in turn, promotes a positive view of staying single. The findings indicate that there may be a rising trend of young people remaining single in the future, potentially due to the growing popularity of the lying flatism lifestyle. To address the potential decline in marriage and birth rates, it is crucial to provide support for the coping mechanisms of the younger generation as they navigate the increasing pressures and demands of modern life and living conditions.

## Data Availability

The raw data, analysis code, and materials used in this study are not openly available but are available upon request to the corresponding author. The data collection and analysis were not pre-registered.
